# Rethinking non-human primate models for emerging viral infections after COVID-19

**DOI:** 10.1186/s42826-026-00299-1

**Published:** 2026-09-24

**Authors:** Dong-Yeon Kim, Jung Joo Hong

**Affiliations:** 1 National Primate Research Centre, Korea Research Institute of Bioscience and Biotechnology (KRIBB), Cheongju, Chungcheongbuk, 28116 Republic of Korea; 2 KRIBB School of Bioscience, Korea University of Science & Technology (UST), Daejeon, 34141 Republic of Korea

**Keywords:** Non-human primates, SARS-CoV-2, New approach methodologies

## Abstract

Non-human primates (NHPs) retain particular value in emerging viral infection research when questions require integrated analysis of systemic viral kinetics, protective immunity, tissue pathology, or longitudinal outcomes. However, their cost, limited availability, specialized husbandry, and ethical constraints preclude their routine use as default screening platforms. Using severe acute respiratory syndrome coronavirus 2 (SARS-CoV-2) as a case study, we compare how rhesus macaques, cynomolgus macaques, African green monkeys, baboons, and marmosets have contributed to studies of pathogenesis, vaccine protection, therapeutic efficacy, immune memory, and post-acute outcomes. We emphasize that these species–question relationships cannot be transferred uncritically to future pathogens; model suitability must be re-established according to receptor usage, tissue tropism, disease phenotype, and experimental objective. AI-guided prediction, human organoids, and organ-on-chip systems can support this process by prioritizing variants and candidates, identifying tissue-specific mechanisms, and refining endpoints before NHP studies begin. We therefore propose a question-driven workflow in which computational and human-relevant platforms narrow the evidence gap before fit-for-purpose NHP validation. This strategy can improve interpretability, reproducibility, and alignment with the 3Rs while preserving NHP use for questions that require intact organism-level biology.

**Graphical Abstract:**

**Figure:**
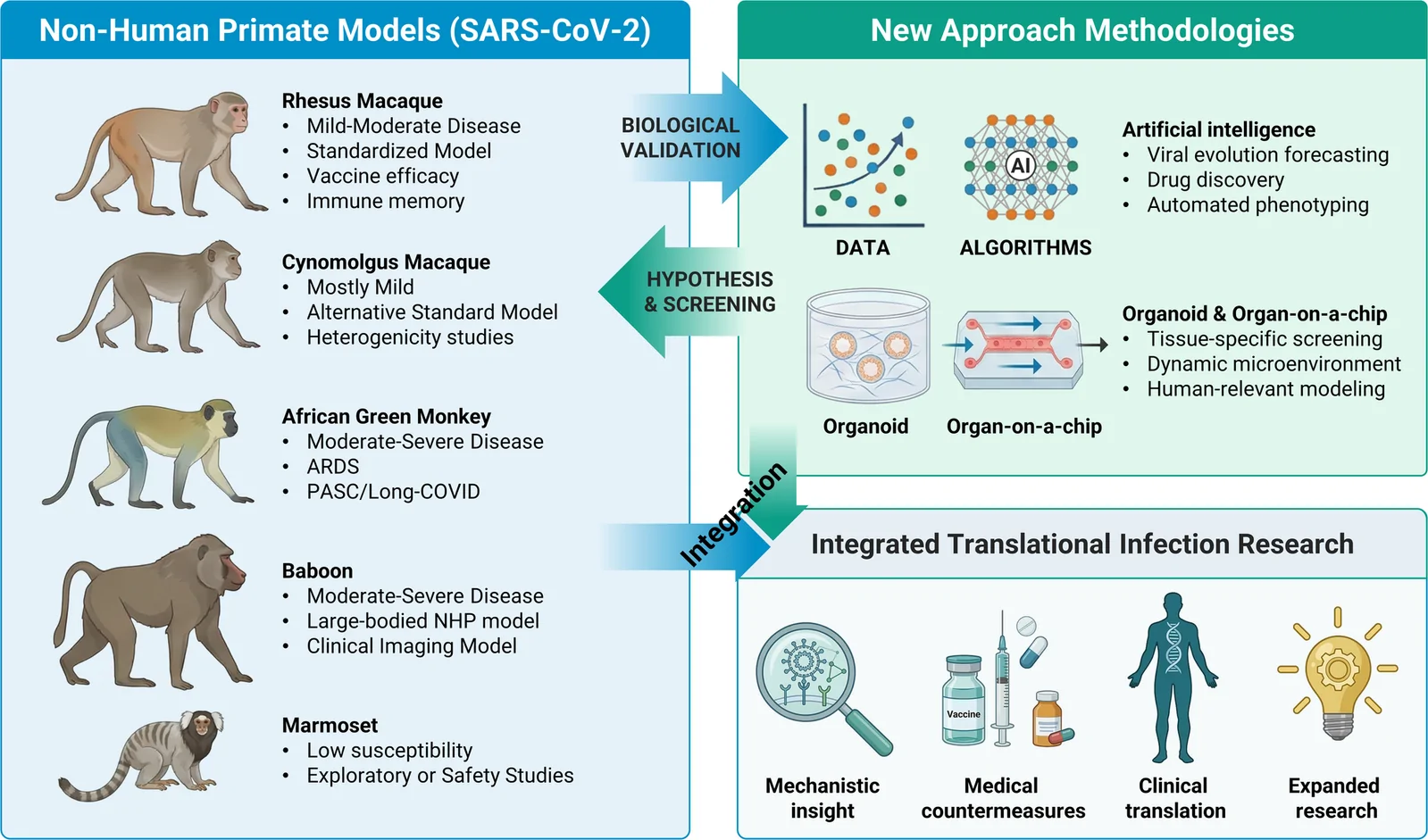

## Background

The continued evolution of severe acute respiratory syndrome coronavirus 2 (SARS-CoV-2) has exposed a central preclinical challenge in infectious disease modeling: the lack of animal systems that are both biologically informative and operationally deployable at outbreak speed [1, 2]. Among the available models, non-human primates (NHPs) remain uniquely valuable because they closely reproduce the integrated virological, immunological, and longitudinal features of human infection [3].

Several key biological and experimental features explain the central role of NHPs in SARS-CoV-2 research. First, phylogenetic proximity between humans and NHPs, reflected in the close structural similarity between human and primate angiotensin-converting enzyme 2 (ACE2), enables productive infection in Old World primates and supports relevant viral entry and tropism [4]. Second, NHPs recapitulate important aspects of human disease pathology, allowing the study of infection dynamics across multiple tissues [3, 5]. Third, their immunological characteristics enable predictive analyses of host immune responses, including protection, durability, and tissue-resident immunity that actively shape local antiviral immunity [3, 6, 7]. Fourth, NHP models provide strong translational relevance, supporting studies of vaccine efficacy, rechallenge, and pharmacological interventions [3]. Fifth, controlled experimental conditions—including defined inoculation, serial bronchoalveolar lavage, advanced imaging, and multi-tissue necropsy—enable mechanistic analyses that are difficult to achieve in patients [3]. In addition, their relatively long lifespan and physiological similarity to humans make NHP models uniquely suited for longitudinal studies of immune memory, enabling direct tracking of disease progression and durable immune responses over extended periods [8]. These advantages have made NHPs indispensable during the acute phase of coronavirus disease 2019 (COVID-19) and continue to support studies of rechallenge, vaccine durability, tissue-resident immunity, and post-acute sequelae.

However, the same experimental control that strengthens internal validity can limit external validity. Defined inoculation routes and doses, synchronized infection times, and comparatively homogeneous cohorts do not fully reproduce the heterogeneous exposure histories, comorbidities, and immune backgrounds of human populations, a limitation also discussed in HIV-1 vaccine research [9, 10]. The role of NHPs is therefore being reconsidered not because their scientific value has diminished, but because their translational limitations, resource requirements, and the changing methodological landscape are reshaping the questions for which they provide the greatest benefit.

This reassessment is being accelerated by converging policy, infrastructure, and methodological changes. The U.S. Food and Drug Administration has launched a roadmap to reduce animal testing through validated new approach methodologies, such as organ-on-chip systems, computational models, and advanced in vitro assays [11]. In parallel, pressures on primate infrastructure, including the phase-out of monkey research at the Centers for Disease Control and Prevention and the Dutch decision to redirect Biomedical Primate Research Centre funding toward alternative methods by 2030, suggest that future infection-model research will depend on using NHPs more strategically rather than more routinely [11–13].

These shifts do not diminish the importance of NHP models; rather, they sharpen the need for their optimal use. Studies in NHPs remain uniquely capable of resolving integrated organism-level processes, such as systemic viral kinetics, tissue pathology, protection correlates, and longitudinal immune memory [14–17]. However, their cost, limited colony availability, specialized husbandry, and ethical constraints restrict scalability at a time when emerging viral threats require rapid prioritization of variants, countermeasures, and tissue-specific mechanisms [3]. These challenges have accelerated the use of new approach methodologies (NAMs), particularly artificial intelligence (AI), organoids, and organ-on-chip systems [18–21].

Accordingly, this Review addresses three linked issues: model selection for defined experimental questions, the growing upstream role of AI-enabled and human-derived platforms, and their integration with targeted NHP validation.

## Main text

### SARS-CoV-2 as a case study for question-driven NHP model selection

SARS-CoV-2 provides a particularly informative case study for re-evaluating how NHP models are selected and deployed in response to an emerging viral infection. The central issue is not the identification of a universally superior species, but the extent to which each model is matched to a specific research question. During the COVID-19 pandemic, NHP studies addressed at least five distinct objectives: pathogenesis and tissue tropism, vaccine efficacy and correlates of protection, therapeutic evaluation, immune memory and reinfection, and post-acute sequelae [3, 4, 15, 22, 23].

To distinguish biological characteristics from question-specific applications, this section is organized into two complementary parts. Part 1 provides a concise orientation to the principal NHP species used in SARS-CoV-2 research, focusing on defining phenotypes, major strengths, and key limitations (Table 1). Part 2 then synthesizes the evidence across species according to the primary experimental objective.

**Table 1:** Comparative strengths of major non-human primate models used in SARS-CoV-2 research

Species	Best-fit experimental role	Major strengths	Main limitations	Key references
Rhesus macaque (*Macaca mulatta*)	Benchmark model for correlates of protection, vaccine/antiviral efficacy, and longitudinal immune analysis	Reproducible respiratory infection; robust humoral and cellular immunity; rechallenge protection; extensive benchmark datasets	Usually models mild-to-moderate rather than critical disease; high cost and limited availability	[14, 24, 25]
Cynomolgus macaque (*Macaca fascicularis*)	Scalable model for mild disease, host heterogeneity, aging, and tissue-resolved analyses	Reproducible viral kinetics; mild/asymptomatic phenotypes; useful for cohort-based and spatial analyses	Limited severe-disease modeling; fewer immunological tools than rhesus macaques	[15, 26]
African green monkey (AGM) (*Chlorocebus aethiops*)	Model for severe inflammatory lung disease, ARDS-like pathology, and longer-term sequelae	Can develop severe pneumonia; strong inflammatory signatures; useful PET/CT and single-cell readouts	Higher inter-individual variability; less standardized and less widely available than macaques	[17, 27]
Baboon (*Papio spp.*)	Targeted option for comorbidity-associated disease and prolonged inflammatory responses	Large body size; age-associated comorbidities; useful vaccine immunogenicity data; prolonged inflammatory phenotype	Fewer standardized protocols and smaller comparative datasets; high maintenance burden	[28, 29]
Marmoset (*Callithrix jacchus*)	Niche or exploratory model rather than a primary efficacy platform	Small-bodied NHP; useful for exploratory or surveillance-oriented contexts	Low susceptibility and limited recapitulation of human COVID-19	[29]

### Species-specific overview

#### Macaque models

The genus *Macaca* comprises several species that are frequently used in biomedical research. Among these, the rhesus macaque (*Macaca mulatta*) and the cynomolgus macaque (*Macaca fascicularis*) have served as the principal macaque models for SARS-CoV-2 research.

#### Rhesus macaque model

Rhesus macaques reproducibly develop high viral loads in the upper and lower respiratory tract following intranasal and intratracheal infection, accompanied by pulmonary infiltrates, interstitial pneumonia, and transient clinical abnormalities that generally resolve within 1–3 weeks [14, 30]. Disease progression can be quantified using standardized clinical scoring, serial thoracic imaging, and histopathological grading of pulmonary inflammation and injury [14, 30]. The model is supported by a mature immunological toolset, including binding and neutralizing antibody assays and functional T-cell analyses, together with one of the most extensive evidence bases among NHP models [2, 14, 30, 31]. A principal limitation is the inconsistent development of severe or fatal disease, as most animals exhibit mild-to-moderate respiratory illness.

#### Cynomolgus macaque model

Cynomolgus macaques exhibit productive viral replication in the upper and lower respiratory tract but predominantly develop mild or asymptomatic disease [15, 26, 32]. Disease severity and inflammatory phenotypes vary with age and metabolic status, making host heterogeneity a potentially informative feature in appropriately designed studies [26]. This variability may support cohort-based study designs in which inter-individual differences are treated as biologically informative rather than solely as experimental confounders. A principal limitation is the inconsistent development of severe, end-stage pathology, and the small cohorts typical of NHP research limit formal analysis of inter-individual variability.

#### African green monkey (AGM) model

AGMs can develop marked inflammatory lung injury, particularly in aged animals, including diffuse alveolar damage and dysregulated pulmonary immune responses [27]. Disease severity appears to be age dependent: young AGMs typically develop self-limited interstitial pneumonia, whereas aged animals may progress to ARDS-like pathology [27]. Serial ^18^F-FDG PET/CT imaging enables sensitive detection of pulmonary inflammation, including subclinical lesions in animals with minimal overt clinical signs [17]. Their main limitations are relatively high inter-individual variability and less extensive standardization than that available for macaque platforms.

#### Baboon model

Baboons (*Papio* spp.) are distinguished by their larger body size and, in some established colonies, the availability of aged animals with naturally occurring comorbidities [28, 33]. They may also exhibit more prolonged pulmonary inflammatory responses than rhesus macaques [29, 34, 35]. Their body size and established use in PET and other imaging-based studies may facilitate imaging-intensive assessments and repeated sampling in longitudinal designs [36, 37]. In SARS-CoV-2 research, these features may support imaging-intensive and longitudinal designs, but direct evidence remains limited. Baboons are therefore potentially informative for age- or comorbidity-associated disease, while their smaller evidence base and fewer widely adopted protocols remain important constraints [28, 29, 34].

#### Marmoset model

Marmosets (*Callithrix jacchus*) show low susceptibility, minimal viral replication, and only transient, mild clinical abnormalities following experimental exposure, with markedly lower permissiveness than Old World primates [29]. Their small body size may offer practical advantages for exploratory studies, but their limited infection and disease phenotypes substantially restrict their value as a primary efficacy or pathogenesis model for SARS-CoV-2.

### Question-driven synthesis

#### Pathogenesis and tissue tropism

Addressing pathogenesis and tissue tropism requires reproducible viral kinetics at defined anatomical sites, a disease-severity range broad enough to capture both self-limited and severe outcomes, and tissue-resolved analysis linked to imaging and histopathology.

Rhesus macaques provide a well-established platform for characterizing respiratory viral kinetics and early innate immune responses. Intranasal and intratracheal inoculation produces consistent viral loads in both the upper and lower respiratory tracts, accompanied by type I interferon responses and pulmonary recruitment of myeloid and lymphoid cells [14, 29, 30]. Cynomolgus macaques show a broadly comparable infection profile, but have been particularly informative for examining host heterogeneity associated with age and metabolic status [15, 26]. Together, these models provide a spectrum from predominantly self-limited respiratory disease in macaques to ARDS-like lung injury in aged African green monkeys (AGMs), enabling comparison of tissue-level immune programs associated with divergent outcomes [27].

The strengths of these models also differ at the level of anatomical resolution. Spatial transcriptomics in cynomolgus macaques has identified microanatomically distinct pulmonary responses [38], while thoracic imaging across macaque models allows radiographic abnormalities to be compared with histopathological findings [14, 30]. AGMs add a more pronounced inflammatory-disease perspective, with strong pulmonary interferon- and IL-6-associated activation, reduced peripheral NK- and T-cell signatures, and temporally regulated responses across lung and lymphoid tissues [39]. Serial ^18^F-FDG PET/CT further enables non-invasive tracking of inflammatory lesions [17].

Model choice therefore depends on whether the primary objective is standardized analysis of respiratory infection and tissue distribution or characterization of a more pronounced inflammatory lung phenotype.

#### Vaccine efficacy and correlates of protection

The suitability of an NHP model for vaccine studies depends on its ability to quantify protection against viral replication, define immune correlates before and after challenge, and assess the breadth and durability of vaccine-induced immunity.

Vaccine studies in rhesus macaques have established quantitative relationships between prechallenge immune responses and subsequent protection. Nucleoside-modified mRNA vaccines induce robust neutralizing antibody and Th1-biased cellular responses and markedly reduce viral replication, particularly in the lower respiratory tract [24]. Neutralizing antibody titers correlate with protection, while passive-transfer studies demonstrate dose-dependent IgG-mediated protection and an additional contribution of T cells when antibody levels are subprotective [40]. These findings make rhesus macaques the best-characterized model for defining correlates of vaccine protection.

Other NHP species extend this evidence base rather than simply reproducing it. Macaque challenge studies have assessed protection against the Omicron variant [41], and cynomolgus experiments have tested an RBD-depleted spike immunogen against antigenically distinct strains [42]. Baboons have contributed primarily to assessments of immunogenicity magnitude and durability: NVX-CoV2373 induced functional antibody responses exceeding those detected in human convalescent sera [43], and an rS-Beta booster elicited strong anamnestic responses one year after primary immunization [44].

For vaccine studies, the key distinction is therefore not disease severity but the type of evidence required: quantitative correlates of protection, variant-specific challenge, or durable immunogenicity.

#### Therapeutic evaluation

For therapeutic studies, the appropriate model depends primarily on the intended mechanism of action. Direct antivirals require reproducible viral kinetics, whereas host-directed or disease-modifying interventions require a measurable inflammatory phenotype.

The consistent respiratory viral kinetics of rhesus macaques support quantitative antiviral assessment. Early remdesivir treatment reduced lung viral loads, pulmonary pathology, and clinical disease severity, directly demonstrating the utility of this model for antiviral efficacy testing [45].

When the therapeutic target is inflammation or tissue injury, AGMs may be more informative than mild-disease macaque models because they can develop a more pronounced, compartmentalized inflammatory phenotype, including pulmonary interferon- and IL-6-associated activation and immune signatures that overlap with severe human disease [27, 39, 46]. However, published studies directly evaluating anti-inflammatory or disease-modifying interventions in SARS-CoV-2-infected AGMs remain limited. Their use for this purpose is therefore supported by biological rationale but is not yet an established application.

The current evidence base is consequently asymmetric: antiviral efficacy has been demonstrated directly in rhesus macaques, whereas host-directed therapies remain comparatively underexplored in models that better reproduce severe inflammatory disease.

#### Immune memory, reinfection, and mucosal immunity

Immune memory studies must address both systemic protection across antigenically distinct variants and local immunity within respiratory tissues and draining lymphoid compartments.

Systemic protection and recall immunity have been demonstrated most directly in rhesus macaques. Primary infection protects against rechallenge and induces rapid anamnestic responses [25, 30], while later studies have characterized cross-variant neutralization, antigenic relationships, and the effects of prior exposure and immune imprinting on recall responses [47–51]. These responses are not restricted to the circulation, as local immune activity has also been detected in respiratory tissues [31].

Studies in cynomolgus macaques have provided particularly detailed resolution of tissue-level immunity. Analyses of respiratory tissues and draining lymph nodes have defined local immune memory after infection [52]. A separate rechallenge study demonstrated effective control of viral replication but transient pneumonia in an elderly cynomolgus macaque despite negative viral PCR, suggesting that recall immunity may not completely prevent immune-mediated pulmonary pathology [26]. Viral clearance in the absence of detectable neutralizing antibodies further supports a role for cellular or other non-neutralizing mechanisms [53].

The available evidence base is therefore complementary rather than interchangeable: rhesus studies are strongest for systemic recall and cross-variant protection, whereas cynomolgus studies provide finer resolution of local immune memory and its protective or pathological consequences.

#### Post-acute sequelae and longitudinal outcomes

Post-acute sequelae cannot be evaluated through acute viral load alone. They require repeated assessment of clinical, imaging, and tissue-level abnormalities, separation of persistent viral signals from immune-mediated injury, and analysis of extrapulmonary outcomes.

Post-acute studies in macaques have detected ongoing viral replication and pathology in pulmonary and extrapulmonary tissues after the acute phase [23]. In rhesus lung samples, SARS-CoV-2-associated foam-cell accumulation with profibrotic and prothrombotic signatures has also been reported, suggesting a potential pathway to persistent tissue injury [54].

Cynomolgus macaques and AGMs contribute different longitudinal strengths. Whole-body immunoPET in cynomolgus macaques enables repeated, non-invasive visualization of anatomically distributed viral antigen or associated tissue signals [55], while nanobody PET tracers can extend monitoring to infection-associated inflammatory changes [56]. AGMs broaden the outcome spectrum beyond the respiratory tract, with persistent hyperglycemia and associated inflammatory signatures reported after infection [57].

Baboons may be suitable for selected longitudinal studies because their body size can facilitate repeated imaging and serial sampling [33, 34, 36, 37]. Nevertheless, direct evidence for SARS-CoV-2 post-acute sequelae in this species remains limited.

Longitudinal research may therefore benefit from combining the mechanistic depth of rhesus studies with the non-invasive imaging and extrapulmonary strengths of cynomolgus macaques and AGMs. Baboons remain a plausible but insufficiently validated option.

Across these major experimental objectives, the low susceptibility and limited disease phenotype of marmosets restrict their utility, leaving them primarily as exploratory or niche models.

### Strategic deployment of primate models

COVID-19 research demonstrated that the value of an NHP model depends on the experimental question rather than on a fixed species hierarchy. Controlled exposure, serial sampling, imaging, and integrated immune analysis enabled decisive studies of rechallenge protection, correlates of protection, vaccine durability, antiviral efficacy, and severe inflammatory phenotypes [3]. However, these capabilities were distributed across models and had to be established empirically during the pandemic.

These scientific advantages do not eliminate the translational limitations of NHP research. Interspecies differences in immune architecture, baseline microbiology, aging, and disease severity constrain generalizability [58, 59]. Similar gaps extend beyond SARS-CoV-2. In NHP dengue models, peak viral loads after experimental inoculation are generally lower than those observed in naturally infected humans, potentially complicating the interpretation of vaccine efficacy [60]. Likewise, simian immunodeficiency virus–based models reproduce selected aspects of human HIV-1 infection [61], but no single macaque model captures the full spectrum of human disease.

Because model suitability often becomes clear only after comparative studies, an NHP-only program may be inefficient for early model qualification and candidate prioritization. During COVID-19, model selection required iterative comparisons across species, challenge designs, host factors, and viral variants, consuming substantial time and resources. Future outbreaks may not permit a comparable process, particularly when receptor usage, tissue tropism, and immune-evasion mechanisms are initially uncertain.

NAMs can reduce this uncertainty before in vivo studies begin. Computational approaches can estimate receptor compatibility, viral evolution, and candidate mechanisms; organoids can test human tissue permissiveness and epithelial responses; and organ-on-chip systems can identify barrier, vascular, or immune interactions that warrant organism-level evaluation. NHP studies can then focus on questions involving systemic protection, integrated inflammatory pathology, and longitudinal outcomes that are not adequately resolved by in vitro or computational systems [58, 62].

This staged logic is applicable beyond SARS-CoV-2 even though the specific species-question relationships established during COVID-19 will not transfer directly to every pathogen. It should be viewed as a decision framework rather than a mandatory linear sequence: some high-consequence pathogens may justify earlier NHP work, whereas other questions may be resolved without NHP use. Its broader value lies in narrowing the biological questions and endpoints that justify NHP investigation, rather than predetermining which species must be used.

Among the diverse NAMs platforms, AI and computational modeling, human organoids, and organ-on-chip systems are particularly relevant to emerging viral infection research. The following sections examine their complementary capabilities and how these can be integrated into specific stages of NHP research.

### AI and computational modeling

AI has become an increasingly important upstream tool in infectious disease research because it can integrate genomic, structural, and epidemiological data at scales that cannot be explored experimentally [63]. In SARS-CoV-2, machine-learning and deep-learning models have been used to predict mutational fitness, immune escape, and candidate future sequences, enabling prioritization of variants and antigens before they become dominant in the population [64–69].

AI is transforming therapeutic discovery by enabling efficient exploration of complex design spaces. Approaches such as drug-repurposing models, structure-guided antibody design, AlphaFold-enabled workflows, and multi-agent virtual laboratory systems reduce experimental search spaces and facilitate rapid candidate optimization under human supervision [68, 69]. These approaches are particularly useful when large mutational or chemical design spaces make conventional empirical screening impractical.

A second frontier, of particular relevance to infection-model studies, is the application of AI-enabled phenotyping within animal experiments. Techniques such as computer vision, markerless pose estimation, automated identification, and deep learning–based image analysis enable the conversion of qualitative observations into standardized digital phenotypes, thereby enhancing the objectivity, reproducibility, and scalability of NHP studies [70–73]. This approach is particularly valuable in infection models, where subtle changes in activity, posture, mobility, and social behavior may influence biologically meaningful signals that are not captured by virological measurements alone.

However, AI is constrained by training-data quality, domain shift, incomplete biological annotation, and the interpretability limits of black-box models [74, 75]. Computational predictions also lack the tissue context and coordinated physiological responses required to assess complete disease trajectories. The principal value of AI in infection-model research is therefore to prioritize hypotheses, variants, and experimental candidates for evaluation in systems that preserve the relevant level of biological complexity.

### Organoid and organ-on-chip systems

Human-derived organoids have become central NAM platforms for infection biology because they preserve three-dimensional tissue architecture and cellular diversity while enabling controlled experimental infection in human-relevant systems [76, 77]. Respiratory, intestinal, neural, and other organoid systems have been used to define viral tropism, compare variant replication, resolve epithelial antiviral responses, and screen therapeutic candidates under conditions that are more physiologically informative than standard monolayer culture [76, 77].

Organoids are especially well suited for investigating early tissue-specific processes, such as cellular tropism, epithelial barrier function, activation of innate immune pathways, and the influence of host genetic background on tissue-level responses [76, 77]. Their principal strength lies in resolving human tissue biology that may not be reproduced consistently across animal species.

Microphysiological systems, including organ-on-chip platforms, extend the utility of organoids by introducing microfluidic flow, tissue–tissue interfaces, immune-cell recruitment, and mechanical cues that are not captured in conventional static organoid cultures [20]. Different platforms can address distinct aspects of viral pathogenesis. Lung-on-chip systems model respiratory entry, epithelial–endothelial barrier injury, alveolar inflammation, and immune-cell recruitment [78], whereas gut-on-chip models enable investigation of enteric tropism and epithelial permeability [79]. Vascular and blood–brain barrier chips can be used to examine endothelial dysfunction, barrier injury, and neuroinflammation, while interconnected multi-organ platforms provide opportunities to study organ–organ communication and extrapulmonary consequences of infection [80]. Related immune-mimetic systems provide a complementary approach. The Modular Immune In vitro Construct (MIMIC) platform uses human donor-derived immune cells and modular tissue-equivalent compartments to reproduce selected features of innate and adaptive immune responses [81]. Such systems have been used to evaluate cytokine responses, T-cell activation, and antibody production following antigenic stimulation [81, 82]. Lymphoid-follicle-on-chip platforms further reproduce aspects of lymphoid organization and vaccination-induced antibody responses in a microfluidic environment [83]. Together, these platforms extend NAM-based infection research beyond epithelial tropism to include vascular, neurological, systemic, and immune-response components.

Important technical constraints remain. Most organoids incompletely reproduce vascularization, adaptive immunity, and long-term multicellular coordination, although engineered systems are beginning to address some of these limitations [84]. Organ-on-chip platforms remain technically demanding and are not yet standardized consistently across laboratories [84]. These constraints define their most appropriate role in infection research: resolving human tissue-specific mechanisms and identifying candidate processes for subsequent evaluation at higher levels of biological organization.

### Linking NAMs to specific stages of NHP research

The practical value of NAMs lies in the specific decisions they inform during NHP study development. Their contributions can be organized across model qualification, experimental design, candidate prioritization, and endpoint selection.

At the model-qualification stage, AI-based analyses can provide initial estimates of viral evolution, antigenic and mutational change, host–pathogen interactions, and candidate disease mechanisms [85]. Human-derived organoids can then test whether the predicted host–pathogen interactions result in cellular permissiveness, tissue-specific replication, or characteristic host responses [76, 77]. Together, these findings can inform whether direct NHP evaluation is biologically justified and which anatomical compartments should be prioritized during model development. A similar approach could be applied to newly emerging pathogens. For example, human airway organoids could provide initial evidence of respiratory permissiveness and replication during a Nipah virus outbreak [86], helping to guide the selection of tissues and endpoints for subsequent NHP model qualification.

NAM-derived findings can also refine NHP experimental design. Organoid analyses may guide the selection of target tissues, sampling windows, and virological or immunological endpoints by identifying where and when infection-associated responses are most likely to occur. Organ-on-chip findings can similarly indicate whether vascular, barrier, or inflammatory mechanisms should be incorporated into the in vivo study design. For example, complement-mediated endothelial injury identified in human vascular organoids was subsequently examined in SARS-CoV-2-infected cynomolgus macaques, illustrating how a mechanism identified in a human-derived system can guide targeted in vivo assessment [87].

For vaccine and therapeutic studies, AI and human-relevant platforms can be used to prioritize variants, antigens, antibodies, or drug candidates before NHP resources are committed. Rather than testing a broad range of candidates across multiple animal cohorts, NHP studies can then focus on a smaller number of well-supported hypotheses requiring integrated assessment of systemic immunity, protection, safety, or longitudinal outcome.

NAMs can also be combined with associated analytical platforms. Host-factor screening and spatial or single-cell profiling can identify dependency factors and tissue-state signatures that can subsequently be measured in NHP blood or tissues, allowing cross-platform assessment of whether the same biological signatures are preserved at the organism level [88].

Together, these applications allow NHP studies to begin with a narrower, biologically supported question and a predefined set of tissues, sampling points, endpoints, and progression criteria. This approach can reduce exploratory animal use while improving the interpretability and translational value of the studies that proceed. Table 2 summarizes how each platform can inform candidate prioritization, study design, endpoint selection, and subsequent NHP evaluation.

**Table 2:** How major NAM platforms complement non-human primate studies in emerging viral infection research

Platform	Best use in viral infection research	Representative outputs	Key limitations	Best interface with NHP studies	Selected references
AI-driven computational modeling	Variant prioritization, immune-escape forecasting, candidate triage, automated phenotyping	Predicted fitness, antigen ranking, drug/antibody prioritization, digital behavioral or imaging readouts	Depends on training-data quality; limited interpretability; cannot reproduce tissue context or integrated immunity	Narrows the search space before animal studies and enhances digital phenotyping within NHP experiments	[64, 68, 69, 89–100]
Human organoids	Tissue tropism, epithelial response, human cell susceptibility, antiviral screening	Cell-type tropism, replication kinetics, innate immune responses, patient-specific variability	Incomplete vascular, immune, and systems-level complexity	Resolves human tissue mechanisms before targeted in vivo validation	[19, 76, 77, 87, 101–103]
Organ-on-chip / microphysiological systems	Dynamic barrier modeling, immune-cell recruitment, interface biology, flow-dependent injury	Barrier disruption, cytokine injury, tissue-tissue interactions, drug response under microfluidic conditions	Technically demanding; lower throughput; incomplete standardization across laboratories	Tests mechanistic hypotheses under dynamic human-relevant conditions before NHP escalation	[20, 104–106]
Advanced screening and multi-omics platforms	Host-factor discovery, compound screening, spatial and single-cell infection profiling	Antiviral hits, host dependency factors, tissue-state maps, cell-state transitions	Usually capture only partial biological scales and require downstream validation	Provides mechanistic depth that can be aligned with NHP tissue and blood phenotypes	[88]

### Standardization and reproducibility

The practical success of staged integration depends not only on the capabilities of each platform, but also on whether transitions between platforms are defined and reported transparently. Preclinical studies should report the experimental question, model-selection rationale, inoculum identity, infection route, sampling schedule, primary endpoints, and go/no-go criteria in a form that allows comparison across species and platforms.

This requirement is particularly important when computational and experimental platforms are incorporated into the same workflow. A variant prioritized by computational modeling may fail to replicate in airway organoids, and a candidate that performs well in a chip system may not produce the expected effect in NHPs. Such discrepancies should be treated as decision-relevant findings rather than simple experimental failures. Investigators should therefore define the intended purpose of each platform and the criteria used to advance, modify, or discontinue a candidate [19].

**Fig. 1:**
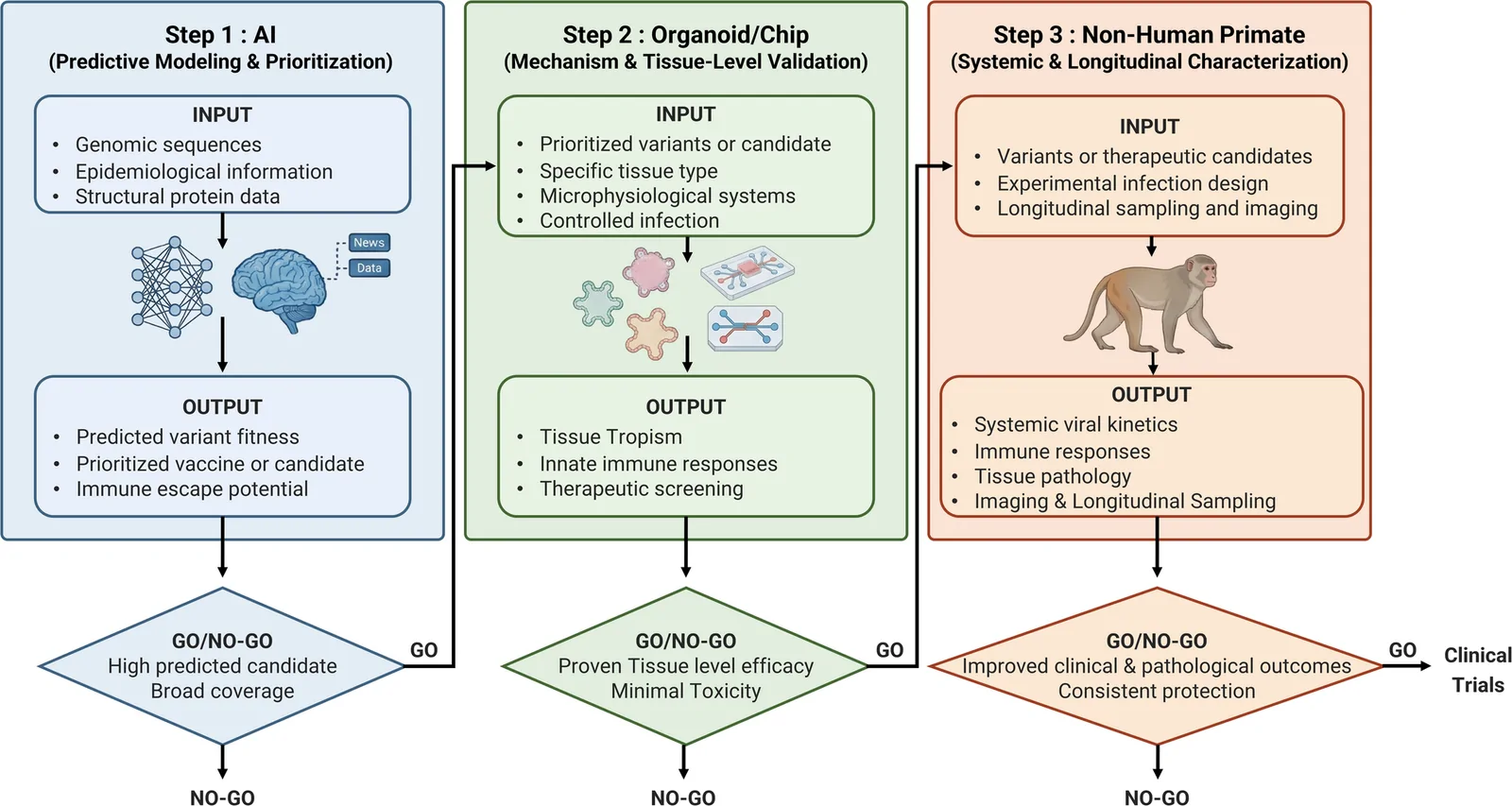
AI-enabled translational workflow linking computational prioritization, human-relevant mechanistic platforms, and targeted non-human primate validation. This schematic diagram presents a staged framework for emerging viral infection research. Step 1 uses AI-enabled modeling to prioritize variants, antigens, or therapeutic candidates from genomic, structural, and epidemiological inputs. Step 2 uses organoid and organ-on-chip systems to test tissue tropism, barrier biology, innate immune responses, and early therapeutic activity in human-relevant settings. Step 3 uses targeted non-human primate studies for organism-level validation of high-priority candidates through longitudinal sampling, imaging, pathology, and systemic immune analysis. Go/no-go decisions at each stage guide progression toward clinical translation

From a 3Rs (Replacement, Reduction, and Refinement) perspective, the contribution of AI and human-relevant NAMs should be judged by whether they support clearer go/no-go decisions, better-defined endpoints, and more informative use of NHP cohorts. Figure 1 proposes a practical staged framework in which AI-based prioritization and human-relevant NAMs progressively inform targeted NHP evaluation through predefined go/no-go decisions.

## Conclusions

Infectious disease research is moving toward platform integration rather than reliance on a single experimental system, since no individual model can simultaneously capture viral evolution, human tissue tropism, coordinated immunity, and longitudinal disease outcomes [107, 108]. As illustrated by SARS-CoV-2, this integration is best achieved through a staged translational pipeline in which model selection is guided by the specific experimental question rather than a fixed species hierarchy: computational and human-derived systems address candidate prioritization and tissue-specific mechanisms upstream, while NHP studies are reserved selectively for questions involving systemic protection, integrated pathology, and longitudinal outcomes that require intact organism-level biology.

The species–question relationships identified during COVID-19 should not be transferred directly to future emerging pathogens. Model suitability must instead be re-established according to pathogen-specific receptor usage, tissue tropism, disease phenotype, and experimental objective. Rethinking NHP models after COVID-19 therefore means translating the lessons and limitations of the pandemic into a flexible decision framework in which computational and human-relevant platforms support timely, fit-for-purpose, and better-justified NHP studies.

## Data Availability

No datasets were generated or analysed during the current study.

## Abbreviations

ACE2: Angiotensin-converting enzyme 2
AGM: African green monkeys
AI: Artificial intelligence
COVID-19: Coronavirus disease 2019
NAMs: New approach methodologies
NHPs: Non-human primates
SARS-CoV-2: Severe acute respiratory syndrome coronavirus 2

## References

[1] Holmes EC. The emergence and evolution of SARS-CoV-2. Annu Rev Virol. 2024;11:21–42. 10.1146/annurev-virology-093022-013037 38631919

[2] Chen Z, Yuan Y, Hu Q, Zhu A, Chen F, Li S, et al. SARS-CoV-2 immunity in animal models. Cell Mol Immunol. 2024;21:119–33. 10.1038/s41423-023-01122-w PMC10806257 38238440

[3] Trichel AM. Overview of nonhuman primate models of SARS-CoV-2 infection. Comp Med. 2021;71:411–32. 10.30802/AALAS-CM-20-000119 PMC8594265 34548126

[4] Damas J, Hughes GM, Keough KC, Painter CA, Persky NS, Corbo M, et al. Broad host range of SARS-CoV-2 predicted by comparative and structural analysis of ACE2 in vertebrates. Proc Natl Acad Sci U S A. 2020;117:22311–22. 10.1073/pnas.2010146117 PMC7486773 32826334

[5] Estes JD, Wong SW, Brenchley JM. Nonhuman primate models of human viral infections. Nat Rev Immunol. 2018;18:390–404. 10.1038/s41577-018-0005-7 PMC5970954 29556017

[6] Wei X, Rong N, Liu J. Prospects of animal models and their application in studies on adaptive immunity to SARS-CoV-2. Front Immunol. 2022;13:993754. 10.3389/fimmu.2022.993754 PMC9523127 36189203

[7] Joag V, Bimber BN, Quarnstrom CF, Bollimpelli VS, Schenkel JM, Fraser KA, et al. Primate resident memory T cells activate humoral and stromal immunity. Immunity. 2025;58:2541–e25556. 10.1016/j.immuni.2025.09.009 PMC12614499 41092897

[8] Meyer C, Kerns A, Haberthur K, Messaoudi I. Improving immunity in the elderly: current and future lessons from nonhuman primate models. Age (Dordr). 2012;34:1157–68. 10.1007/s11357-011-9353-y PMC3448983 22180097

[9] Klasse PJ, Moore JP. Reappraising the value of HIV-1 vaccine correlates of protection analyses. J Virol. 2022;96:e00034–22. 10.1128/jvi.00034-22 PMC9044961 35384694

[10] Kelkar NS, Carpenter MC, Ackerman ME. Losing less in translation: relating the preclinical evidence base for active and passive immunity to prevention of HIV-1 infection in humans. J Med Primatol. 2025;54:e70040. 10.1111/jmp.70040 PMC13162053 41107675

[11] US Food and Drug Administration. Roadmap to reducing animal testing in preclinical safety studies. Silver Spring (MD): US Food and Drug Administration; 2025. pp. 1–11.

[12] Grimm D. Political appointee tells CDC to end monkey research. Science. 2025;390:868–9. 10.1126/science.aee1366 41308139

[13] Bijker WE, Geluk A, van Gool P, Krabbenborg L, Leufkens B, Meijboom F, et al. Research involving non-human primates: four policy scenarios for the Netherlands: report by the Committee on Research Involving Non-Human Primates. The Hague: Ministerie van OCW; 2025.

[14] Munster VJ, Feldmann F, Williamson BN, van Doremalen N, Pérez-Pérez L, Schulz J, et al. Respiratory disease in rhesus macaques inoculated with SARS-CoV-2. Nature. 2020;585:268–72. 10.1038/s41586-020-2324-7 PMC7486227 32396922

[15] Rockx B, Kuiken T, Herfst S, Bestebroer T, Lamers MM, Oude Munnink BB, et al. Comparative pathogenesis of COVID-19, MERS, and SARS in a nonhuman primate model. Science. 2020;368:1012–5. 10.1126/science.abb7314 PMC7164679 32303590

[16] Lu S, Zhao Y, Yu W, Yang Y, Gao J, Wang J, et al. Comparison of nonhuman primates identified the suitable model for COVID-19. Signal Transduct Target Ther. 2020;5:157. 10.1038/s41392-020-00269-6 PMC7434851 32814760

[17] Hartman AL, Nambulli S, McMillen CM, White AG, Tilston-Lunel NL, Albe JR, et al. SARS-CoV-2 infection of African green monkeys results in mild respiratory disease discernible by PET/CT imaging and shedding of infectious virus from both respiratory and gastrointestinal tracts. PLoS Pathog. 2020;16:e1008903. 10.1371/journal.ppat.1008903 PMC7535860 32946524

[18] Theodosiou AA, Read RC. Artificial intelligence, machine learning and deep learning: potential resources for the infection clinician. J Infect. 2023;87:287–94. 10.1016/j.jinf.2023.07.006 37468046

[19] Han Y, Yang L, Lacko LA, Chen S. Human organoid models to study SARS-CoV-2 infection. Nat Methods. 2022;19:418–28. 10.1038/s41592-022-01453-y 35396481

[20] Alonso-Roman R, Mosig AS, Figge MT, Papenfort K, Eggeling C, Schacher FH, et al. Organ-on-chip models for infectious disease research. Nat Microbiol. 2024;9:891–904. 10.1038/s41564-024-01645-6 38528150

[21] Hammel JH, Zatorski JM, Cook SR, Pompano RR, Munson JM. Engineering in vitro immune-competent tissue models for testing and evaluation of therapeutics. Adv Drug Deliv Rev. 2022;182:114111. 10.1016/j.addr.2022.114111 PMC8908413 35031388

[22] Choudhary S, Kanevsky I, Yildiz S, Sellers RS, Swanson KA, Franks T, et al. Modeling SARS-CoV-2: comparative pathology in rhesus macaque and golden Syrian hamster models. Toxicol Pathol. 2022;50:280–93. 10.1177/01926233211072767 PMC8819578 35128980

[23] Böszörményi KP, Stammes MA, Fagrouch ZC, Kiemenyi-Kayere G, Niphuis H, Mortier D, et al. The post-acute phase of SARS-CoV-2 infection in two macaque species is associated with signs of ongoing virus replication and pathology in pulmonary and extrapulmonary tissues. Viruses. 2021;13:1673. 10.3390/v13081673 PMC8402919 34452537

[24] Vogel AB, Kanevsky I, Che Y, Swanson KA, Muik A, Vormehr M, et al. BNT162b vaccines protect rhesus macaques from SARS-CoV-2. Nature. 2021;592:283–9. 10.1038/s41586-021-03275-y 33524990

[25] Chandrashekar A, Liu J, Martinot AJ, McMahan K, Mercado NB, Peter L, et al. SARS-CoV-2 infection protects against rechallenge in rhesus macaques. Science. 2020;369:812–7. 10.1126/science.abc4776 PMC7243369 32434946

[26] Urano E, Okamura T, Ono C, Ueno S, Nagata S, Kamada H, et al. COVID-19 cynomolgus macaque model reflecting human COVID-19 pathological conditions. Proc Natl Acad Sci U S A. 2021;118:e2104847118. 10.1073/pnas.2104847118 PMC8639365 34625475

[27] Blair RV, Vaccari M, Doyle-Meyers LA, Roy CJ, Russell-Lodrigue K, Fahlberg M, et al. Acute respiratory distress in aged, SARS-CoV-2-infected African green monkeys but not rhesus macaques. Am J Pathol. 2021;191:274–82. 10.1016/j.ajpath.2020.10.016 PMC7648506 33171111

[28] Chang MC, Hild S, Grieder F. Nonhuman primate models for SARS-CoV-2 research: consider alternatives to macaques. Lab Anim (NY). 2021;50:113–4. 10.1038/s41684-021-00755-6 33785923

[29] Singh DK, Singh B, Ganatra SR, Gazi M, Cole J, Thippeshappa R, et al. Responses to acute infection with SARS-CoV-2 in the lungs of rhesus macaques, baboons and marmosets. Nat Microbiol. 2021;6:73–86. 10.1038/s41564-020-00841-4 PMC7890948 33340034

[30] Deng W, Bao L, Liu J, Xiao C, Liu J, Xue J, et al. Primary exposure to SARS-CoV-2 protects against reinfection in rhesus macaques. Science. 2020;369:818–23. 10.1126/science.abc5343 PMC7402625 32616673

[31] Zheng H, Chen Y, Li J, Li H, Zhao X, Li J, et al. Longitudinal analyses reveal distinct immune response landscapes in lung and intestinal tissues from SARS-CoV-2-infected rhesus macaques. Cell Rep. 2022;39:110864. 10.1016/j.celrep.2022.110864 PMC9080054 35594870

[32] Li Y, Shi J, Xia J, Duan J, Chen L, Yu X, et al. Asymptomatic and symptomatic patients with non-severe coronavirus disease (COVID-19) have similar clinical features and virological courses: a retrospective single center study. Front Microbiol. 2020;11:1570. 10.3389/fmicb.2020.01570 PMC7344298 32754137

[33] Cox LA, Comuzzie AG, Havill LM, Karere GM, Spradling KD, Mahaney MC, et al. Baboons as a model to study genetics and epigenetics of human disease. ILAR J. 2013;54:106–21. 10.1093/ilar/ilt038 PMC3924757 24174436

[34] Kwon T. Utilizing non-human primate models to combat recent COVID-19/SARS-CoV-2 and viral infectious disease outbreaks. J Med Primatol. 2024;53:e12689. 10.1111/jmp.12689 38084001

[35] Casel MAB, Rollon RG, Choi YK. Experimental animal models of coronavirus infections: strengths and limitations. Immune Netw. 2021;21:e12. 10.4110/in.2021.21.e12 PMC8099610 33996168

[36] Szabó CA, Narayana S, Kochunov PV, Franklin C, Knape K, Davis MD, et al. PET imaging in the photosensitive baboon: case-controlled study. Epilepsia. 2007;48:245–53. 10.1111/j.1528-1167.2006.00949.x 17295617

[37] Szabo CA, Salinas FS. Neuroimaging in the epileptic baboon. Front Vet Sci. 2022;9:908801. 10.3389/fvets.2022.908801 PMC9330034 35909685

[38] Oh T, Kim G, Baek SH, Woo Y, Koo BS, Hwang EH, et al. Spatial transcriptome atlas reveals pulmonary microstructure-specific COVID-19 gene signatures in cynomolgus macaques. Commun Biol. 2023;6:879. 10.1038/s42003-023-05253-8 PMC10462721 37640792

[39] Woolsey C, Borisevich V, Prasad AN, Agans KN, Deer DJ, Dobias NS, et al. Establishment of an African green monkey model for COVID-19 and protection against re-infection. Nat Immunol. 2021;22:86–98. 10.1038/s41590-020-00835-8 PMC7790436 33235385

[40] McMahan K, Yu J, Mercado NB, Loos C, Tostanoski LH, Chandrashekar A, et al. Correlates of protection against SARS-CoV-2 in rhesus macaques. Nature. 2021;590:630–4. 10.1038/s41586-020-03041-6 PMC7906955 33276369

[41] Chandrashekar A, Yu J, McMahan K, Jacob-Dolan C, Liu J, He X, et al. Vaccine protection against the SARS-CoV-2 Omicron variant in macaques. Cell. 2022;185:1549–e155511. 10.1016/j.cell.2022.03.024 PMC8926910 35427477

[42] Letscher H, Guilligay D, Effantin G, Amen A, Sulbaran G, Burger JA, et al. RBD-depleted SARS-CoV-2 spike generates protective immunity in cynomolgus macaques. NPJ Vaccines. 2025;10:63. 10.1038/s41541-025-01113-0 PMC11955555 40159504

[43] Tian JH, Patel N, Haupt R, Zhou H, Weston S, Hammond H, et al. SARS-CoV-2 spike glycoprotein vaccine candidate NVX-CoV2373 immunogenicity in baboons and protection in mice. Nat Commun. 2021;12:372. 10.1038/s41467-020-20653-8 PMC7809486 33446655

[44] Logue J, Johnson RM, Patel N, Zhou B, Maciejewski S, Foreman B, et al. Immunogenicity and protection of a variant nanoparticle vaccine that confers broad neutralization against SARS-CoV-2 variants. Nat Commun. 2023;14:1130. 10.1038/s41467-022-35606-6 PMC9972327 36854666

[45] Williamson BN, Feldmann F, Schwarz B, Meade-White K, Porter DP, Schulz J, et al. Clinical benefit of remdesivir in rhesus macaques infected with SARS-CoV-2. Nature. 2020;585:273–6. 10.1038/s41586-020-2423-5 PMC7486271 32516797

[46] Speranza E, Williamson BN, Feldmann F, Sturdevant GL, Pérez-Pérez L, Meade-White K, et al. Single-cell RNA sequencing reveals SARS-CoV-2 infection dynamics in lungs of African green monkeys. Sci Transl Med. 2021;13:eabe8146. 10.1126/scitranslmed.abe8146 PMC7875333 33431511

[47] Malewana RD, Stalls V, May A, Lu X, Martinez DR, Schäfer A, et al. Nonstabilized SARS-CoV-2 spike mRNA vaccination induces broadly neutralizing antibodies in nonhuman primates. Sci Transl Med. 2025;17:eadn5651. 10.1126/scitranslmed.adn5651 PMC13075761 40498855

[48] Wang S, Sun H, Wang Y, Wang Z, Yuan L, Guo H, et al. Broad neutralizing antibody response of a monomeric spike-based SARS-CoV-2 bivalent vaccine against diverse variants. Proc Natl Acad Sci U S A. 2025;122:e2503254122. 10.1073/pnas.2503254122 PMC12415226 40854137

[49] Rössler A, Netzl A, Lasrado N, Chaudhari J, Mühlemann B, Wilks SH, et al. Nonhuman primate antigenic cartography of SARS-CoV-2. Cell Rep. 2025;44:115140. 10.1016/j.celrep.2024.115140 PMC11781863 39754717

[50] Lenart K, Feuerstein H, Prado Marmorato M, Perez Vidakovics L, McInerney G, Guebre-Xabier M, et al. Coordinated early immune response in the lungs is required for effective control of SARS-CoV-2 replication. Nat Commun. 2025;16:5390. 10.1038/s41467-025-60885-0 PMC12198374 40562777

[51] Case JB, Jain S, Suthar MS, Diamond MS. SARS-CoV-2: the interplay between evolution and host immunity. Annu Rev Immunol. 2025;43:29–55. 10.1146/annurev-immunol-083122-043054 PMC13456083 39705164

[52] Kim DY, Kim G, Oh T, Woo Y, Koo BS, Baek SH, et al. Structural and functional characteristics of local immune memory formation in SARS-CoV-2-infected cynomolgus macaques. Clin Immunol. 2025;281:110589. 10.1016/j.clim.2025.110589 40882689

[53] Ishigaki H, Nakayama M, Kitagawa Y, Nguyen CT, Hayashi K, Shiohara M, et al. Neutralizing antibody-dependent and -independent immune responses against SARS-CoV-2 in cynomolgus macaques. Virology. 2021;554:97–105. 10.1016/j.virol.2020.12.013 PMC7771262 33412411

[54] Battaglia DM, Post CE, Yao W, Wahl A, Gralinski LE, Liu H, et al. SARS-CoV-2 infection induces pro-fibrotic and pro-thrombotic foam cell formation. Nat Microbiol. 2025;10:2616–30. 10.1038/s41564-025-02090-9 PMC12711563 40846999

[55] Detrille A, Huvelle S, van Gils MJ, Geara T, Pascal Q, Snitselaar J, et al. Whole-body visualization of SARS-CoV-2 biodistribution in vivo by immunoPET imaging in non-human primates. Nat Commun. 2025;16:2816. 10.1038/s41467-025-58173-y PMC11928647 40118860

[56] Koopman G, Verhoeven T, Mooij P, Acar RF, Harmand T, Flanagan L, et al. Imaging the immune sequelae of infection with SARS-CoV-2 in nonhuman primates by using two nanobody PET-tracers. J Med Virol. 2024;96:e29956. 10.1002/jmv.29956 39400953

[57] Palmer CS, Perdios C, Abdel-Mohsen M, Mudd J, Datta PK, Maness NJ, et al. Non-human primate model of long-COVID identifies immune associates of hyperglycemia. Nat Commun. 2024;15:6664. 10.1038/s41467-024-50339-4 PMC11335872 39164284

[58] Cauvin AJ, Peters C, Brennan F. Advantages and limitations of commonly used nonhuman primate species in research and development of biopharmaceuticals. In: Bluemel J, Korte S, Schenck E, Weinbauer GF, editors. The Nonhuman Primate in Nonclinical Drug Development and Safety Assessment. San Diego: Academic; 2015. pp. 379–95.

[59] Ishigaki H, Itoh Y. Translational research on pandemic virus infection using nonhuman primate models. Virology. 2025;606:110511. 10.1016/j.virol.2025.110511 40139071

[60] Barban V, Mantel N, De Montfort A, Pagnon A, Pradezynski F, Lang J, et al. Improvement of the dengue virus (DENV) nonhuman primate model via a reverse translational approach based on dengue vaccine clinical efficacy data against DENV-2 and – 4. J Virol. 2018;92:e00440–18. 10.1128/JVI.00440-18 PMC5974474 29593041

[61] Sharma M, Nag M, Del Prete GQ. Minimally modified HIV-1 infection of macaques: development, utility, and limitations of current models. Viruses. 2024;16:1618. 10.3390/v16101618 PMC11512399 39459950

[62] Elfatimi E, Lekbach Y, Prakash S, Karan S, Dorotta JC, Garcia A, et al. Artificial intelligence-, organoid-, and organ-on-chip-powered models to improve pre-clinical animal testing of vaccines and immunotherapeutics: potential, progress, and challenges. Front Artif Intell. 2025;8:1681106. 10.3389/frai.2025.1681106 PMC12615408 41244612

[63] Kraemer MUG, Tsui JL, Chang SY, Lytras S, Khurana MP, Vanderslott S, et al. Artificial intelligence for modelling infectious disease epidemics. Nature. 2025;638:623–35. 10.1038/s41586-024-08564-w PMC11987553 39972226

[64] Starr TN, Greaney AJ, Hilton SK, Ellis D, Crawford KHD, Dingens AS, et al. Deep mutational scanning of SARS-CoV-2 receptor binding domain reveals constraints on folding and ACE2 binding. Cell. 2020;182:1295–e131020. 10.1016/j.cell.2020.08.012 PMC7418704 32841599

[65] Lalmuanawma S, Hussain J, Chhakchhuak L. Applications of machine learning and artificial intelligence for COVID-19 (SARS-CoV-2) pandemic: a review. Chaos Solitons Fractals. 2020;139:110059. 10.1016/j.chaos.2020.110059 PMC7315944 32834612

[66] Ding X, Shang B, Xie C, Xin J, Yu F. Artificial intelligence in the COVID-19 pandemic: balancing benefits and ethical challenges in China’s response. Humanit Soc Sci Commun. 2025;12:245.

[67] Bhat AR, Ahmed S. Artificial intelligence (AI) in drug design and discovery: a comprehensive review. Silico Res Biomed. 2025;1:100049.

[68] Beck BR, Shin B, Choi Y, Park S, Kang K. Predicting commercially available antiviral drugs that may act on the novel coronavirus (SARS-CoV-2) through a drug-target interaction deep learning model. Comput Struct Biotechnol J. 2020;18:784–90. 10.1016/j.csbj.2020.03.025 PMC7118541 32280433

[69] Ke YY, Peng TT, Yeh TK, Huang WZ, Chang SE, Wu SH, et al. Artificial intelligence approach fighting COVID-19 with repurposing drugs. Biomed J. 2020;43:355–62. 10.1016/j.bj.2020.05.001 PMC7227517 32426387

[70] Mathis A, Mamidanna P, Cury KM, Abe T, Murthy VN, Mathis MW, et al. DeepLabCut: markerless pose estimation of user-defined body parts with deep learning. Nat Neurosci. 2018;21:1281–9. 10.1038/s41593-018-0209-y 30127430

[71] Moat GJ, Gaudet-Trafit M, Paul J, Bacardit J, Ben Hamed S, Poirier C. The MacqD deep-learning-based model for automatic detection of socially housed laboratory macaques. Sci Rep. 2025;15:11883. 10.1038/s41598-025-95180-x PMC11977019 40195447

[72] Ye S, Filippova A, Lauer J, Schneider S, Vidal M, Qiu T, et al. SuperAnimal pretrained pose estimation models for behavioral analysis. Nat Commun. 2024;15:5165. 10.1038/s41467-024-48792-2 PMC11192880 38906853

[73] Bala PC, Eisenreich BR, Yoo SBM, Hayden BY, Park HS, Zimmermann J. Automated markerless pose estimation in freely moving macaques with OpenMonkeyStudio. Nat Commun. 2020;11:4560. 10.1038/s41467-020-18441-5 PMC7486906 32917899

[74] Messeri L, Crockett MJ. Artificial intelligence and illusions of understanding in scientific research. Nature. 2024;627:49–58. 10.1038/s41586-024-07146-0 38448693

[75] Rudin C. Stop explaining black box machine learning models for high stakes decisions and use interpretable models instead. Nat Mach Intell. 2019;1:206–15. 10.1038/s42256-019-0048-x PMC9122117 35603010

[76] Dutta D, Clevers H. Organoid culture systems to study host-pathogen interactions. Curr Opin Immunol. 2017;48:15–22. 10.1016/j.coi.2017.07.012 PMC7126332 28756233

[77] Blutt SE, Estes MK. Organoid models for infectious disease. Annu Rev Med. 2022;73:167–82. 10.1146/annurev-med-042320-023055 PMC8887824 34644153

[78] Zhang M, Wang P, Luo R, Wang Y, Li Z, Guo Y, et al. Biomimetic human disease model of SARS-CoV-2-induced lung injury and immune responses on organ chip system. Adv Sci (Weinh). 2021;8:2002928. 10.1002/advs.202002928 PMC7646023 33173719

[79] Guo Y, Luo R, Wang Y, Deng P, Song T, Zhang M, et al. SARS-CoV-2 induced intestinal responses with a biomimetic human gut-on-chip. Sci Bull (Beijing). 2021;66:783–93. 10.1016/j.scib.2020.11.015 PMC7704334 33282445

[80] Wang P, Jin L, Zhang M, Wu Y, Duan Z, Guo Y, et al. Blood-brain barrier injury and neuroinflammation induced by SARS-CoV-2 in a lung-brain microphysiological system. Nat Biomed Eng. 2024;8:1053–68. 10.1038/s41551-023-01054-w 37349391

[81] Higbee RG, Byers AM, Dhir V, Drake D, Fahlenkamp HG, Gangur J, et al. An immunologic model for rapid vaccine assessment–a clinical trial in a test tube. Altern Lab Anim. 2009;37(Suppl 1):19–27. 10.1177/026119290903701S05 19807200

[82] Dauner A, Agrawal P, Salvatico J, Tapia T, Dhir V, Shaik SF, et al. The in vitro MIMIC^®^ platform reflects age-associated changes in immunological responses after influenza vaccination. Vaccine. 2017;35:5487–94. 10.1016/j.vaccine.2017.03.099 28413134

[83] Goyal G, Prabhala P, Mahajan G, Bausk B, Gilboa T, Xie L, et al. Ectopic lymphoid follicle formation and human seasonal influenza vaccination responses recapitulated in an organ-on-a-chip. Adv Sci (Weinh). 2022;9:e2103241. 10.1002/advs.202103241 PMC9109055 35289122

[84] Fan X, Hou K, Liu G, Shi R, Wang W, Liang G. Strategies to overcome the limitations of current organoid technology-engineered organoids. J Tissue Eng. 2025;16:20417314251319475. 10.1177/20417314251319475 PMC12033597 40290859

[85] Odone A, Barbati C, Amadasi S, Schultz T, Resnik DB. Artificial intelligence and infectious diseases: an evidence-driven conceptual framework for research, public health, and clinical practice. Lancet Infect Dis. 2026;26:e152–67. 10.1016/S1473-3099(25)00412-8 40972628

[86] Wälzlein JH, Reusch S, Ospina-Garcia J, Olmer R, Schneider MA, Klotz LV, et al. Human airway organoids as a versatile model to study BSL-4 virus replication and pathogenesis. Sci Rep. 2026;16:10517. 10.1038/s41598-026-45813-6 PMC13035905 41896609

[87] Kawakami E, Saiki N, Yoneyama Y, Moriya C, Maezawa M, Kawamura S, et al. Complement factor D targeting protects endotheliopathy in organoid and monkey models of COVID-19. Cell Stem Cell. 2023;30:1315–e133010. 10.1016/j.stem.2023.09.001 PMC10575686 37802037

[88] Idrees S, Chen H, Sadaf T, Rehman SF, Johansen MD, Paudel KR, et al. Multi-omics to study chronic respiratory diseases and viral infections. Eur Respir Rev. 2026;35:240286. 10.1183/16000617.0286-2024 PMC12801048 41534886

[89] Hie B, Zhong ED, Berger B, Bryson B. Learning the language of viral evolution and escape. Science. 2021;371:284–8. 10.1126/science.abd7331 33446556

[90] Thadani NN, Gurev S, Notin P, Youssef N, Rollins NJ, Ritter D, et al. Learning from prepandemic data to forecast viral escape. Nature. 2023;622:818–25. 10.1038/s41586-023-06617-0 PMC10599991 37821700

[91] Rodriguez-Rivas J, Croce G, Muscat M, Weigt M. Epistatic models predict mutable sites in SARS-CoV-2 proteins and epitopes. Proc Natl Acad Sci U S A. 2022;119:e2113118119. 10.1073/pnas.2113118119 PMC8795541 35022216

[92] Ramachandran A, Lumetta SS, Chen D. PandoGen: generating complete instances of future SARS-CoV-2 sequences using deep learning. PLoS Comput Biol. 2024;20:e1011790. 10.1371/journal.pcbi.1011790 PMC10829978 38241392

[93] Waterhouse A, Bertoni M, Bienert S, Studer G, Tauriello G, Gumienny R, et al. SWISS-MODEL: homology modelling of protein structures and complexes. Nucleic Acids Res. 2018;46:W296–303. 10.1093/nar/gky427 PMC6030848 29788355

[94] Hanack K, Orzeł U, Schloer A, Mehta S, Krishnan A, Filipek S, et al. Structural characterization and AI-enhanced modeling of a broadly neutralizing camelid antibody against SARS-CoV-2 variants. Adv Ther (Weinh). 2026;9:e202500244.

[95] Swanson K, Wu W, Bulaong NL, Pak JE, Zou J. The Virtual Lab of AI agents designs new SARS-CoV-2 nanobodies. Nature. 2025;646:716–23. 10.1038/s41586-025-09442-9 40730228

[96] Jumper J, Evans R, Pritzel A, Green T, Figurnov M, Ronneberger O, et al. Highly accurate protein structure prediction with AlphaFold. Nature. 2021;596:583–9. 10.1038/s41586-021-03819-2 PMC8371605 34265844

[97] Lin Z, Akin H, Rao R, Hie B, Zhu Z, Lu W, et al. Evolutionary-scale prediction of atomic-level protein structure with a language model. Science. 2023;379:1123–30. 10.1126/science.ade2574 36927031

[98] Gawande MS, Zade N, Kumar P, Gundewar S, Weerarathna IN, Verma P. The role of artificial intelligence in pandemic responses: from epidemiological modeling to vaccine development. Mol Biomed. 2025;6:1. 10.1186/s43556-024-00238-3 PMC11695538 39747786

[99] Groff-Vindman CS, Trump BD, Cummings CL, Smith M, Titus AJ, Oye K, et al. The convergence of AI and synthetic biology: the looming deluge. NPJ Biomed Innov. 2025;2:20. 10.1038/s44385-025-00021-1 PMC13055036 42032179

[100] Shahin OR, Ibrahim MN, Alanazi A, Alharithi FS, Alruwaili Y, Alzahrani AA, et al. Predicting genetic evolution of viruses to identify suitable vaccines using artificial intelligence. Sci Rep. 2026;16:5512. 10.1038/s41598-026-35143-y PMC12887023 41634057

[101] Suneesh NS, Bagchi P, Mukherjee A. Organoids gone viral: a comprehensive review on human organoid models to study viral pathogenesis. Viruses. 2026;18:238. 10.3390/v18020238 PMC12944987 41754581

[102] Chu JTS, Lamers MM. Organoids in virology. NPJ Viruses. 2024;2:5. 10.1038/s44298-024-00017-5 PMC11721363 40295690

[103] Li X, Xiao H, Zhou M, Yang C, Yang X, Cheng T, et al. Organoids in respiratory virus research: advances and perspectives. Mol Biomed. 2025;6:100. 10.1186/s43556-025-00343-x PMC12589759 41193785

[104] Ma C, Peng Y, Li H, Chen W. Organ-on-a-chip: a new paradigm for drug development. Trends Pharmacol Sci. 2021;42:119–33. 10.1016/j.tips.2020.11.009 PMC7990030 33341248

[105] Wang Y, Chang X, Deng S, Tang S, Chen P. Functional material probes and advanced technologies in organ-on-a-chip characterization. Theranostics. 2026;16:2488–516. 10.7150/thno.122552 PMC12712907 41424855

[106] Sakthivel V, Dharshan SS, Namasivayam SKR, Arockiaraj J. Advances in 3D organoids and organ-on-a-chip systems for biomedical research. J Biomed Mater Res A. 2026;114:e70028. 10.1002/jbma.70028 41481795

[107] Seal S, Dharmarajan G, Khan I. Evolution of pathogen tolerance and emerging infections: a missing experimental paradigm. Elife. 2021;10:e68874. 10.7554/eLife.68874 PMC8455132 34544548

[108] López-Jiménez AT, Mostowy S. Emerging technologies and infection models in cellular microbiology. Nat Commun. 2021;12:6764. 10.1038/s41467-021-26641-w PMC8604907 34799563

